# Lithium: how low can you go?

**DOI:** 10.1186/s40345-024-00325-y

**Published:** 2024-01-30

**Authors:** Rebecca Strawbridge, Allan H. Young

**Affiliations:** Department of Psychological Medicine, Centre for Affective Disorders, Institute of Psychiatry, Psychology & Neuroscience, King’s College London, 103 Denmark Hill, London, SE5 8AZ UK

In medicine, lithium is best known as the mainstay mood stabiliser for bipolar disorders (Ulrichsen et al. 2023; Yatham et al. 2018) and is also effective in unipolar affective disorders (Scott et al. 2023). Having been investigated for many decades, lithium has additional therapeutic indications both within and outside psychiatry (Bauer and Gitlin 2016). Unlike many other psychotropic medications, lithium is recognised as having a range of neuroprotective effects, including on length of telomeres, hippocampal neurogenesis and reducing peripheral inflammation (Puglisi-Allegra et al. 2021; Strawbridge et al. 2023a). Despite this, lithium is widely reported to be under-used (relative to both the need for this medicine and its evidence base) (Post 2018). Reasons for this are varied, from education of both clinicians (Gomes et al. 2022; Post 2018) and patients (Gomes et al. 2022; Severus et al. 2021) to associated negative beliefs and/or attitudes primarily relating to side effects or safety, again from both clinicians and patients (Hidalgo-Mazzei et al. 2023). Further reasons include complexities or inconveniences related to the need for regular blood monitoring, which appears to be present from patient (McKeown et al. 2022), clinician and service-level perspective (Nikolova et al. 2018). As a result, rates of lithium monitoring adherence are widely suboptimal, which in turn increases the likelihood of adverse effects (Nikolova et al. 2018). Further, it has been reported that initial patient concerns regarding side effects and monitoring are ameliorated after subsequent experience of treatment and monitoring (McKeown et al. 2022).

Lithium dose is certainly relevant when considering its underuse and controversy, with higher doses conferring greater safety issues. Thus, indication is also relevant here as lithium therapeutic dose varies by indication. We discuss here evidence on lithium efficacy and safety at different dose ranges, which are outlined summarily in Table 1.

**Table 1:** Summary of lithium efficacy and safety at different dose ranges

	Serum level / dose range*	Ionic Li dose	Efficacy summary	Safety summary
**‘High’ dose**	> 1.0/1.2mmol/L 0.8-1.0mmol/L	> 250 mg	Severe non-responsive acute mania, aggressive / self-harm behaviour Non-responsive BD if well tolerated	Increased risk of toxicity Long-term: increased safety concerns vs. below
**‘Therapeutic’ dose**	0.6–0.8 mmol/L	~ 112-250 mg	Maintenance BD (also shorter-term depression, mania), rapid cycling BD, suicidality, unipolar depression, possible effect dementia.	Short-term side effects common. Long-term *some* risk to renal +/ thyroid function. Many concerns addressed via monitoring +/ action.
**‘Low dose’**	0.2–0.6 mmol/L	~ 20-100 mg	Possible effect dementia; possible effects as adjunct in depression, mania, limited evidence for other neuropsychiatric benefits.	Generally well tolerated, attenuated concern relative to above.
**‘Micro’ dose**	Unknown	~ 5-20 mg	Limited evidence for effects on mood, anxiety and dementia over long periods.	No known safety concerns.
**‘Trace’ dose**	< 0.1mmol/L	< 5 mg	Possible small effect over long periods- suicide, dementia, ?homicide.	No safety concerns

* serum concentrations 10–14 h after most recent dose, optimally at 12 h (most stable), when using standard immediate release formulations (daily peak concentrations up to 3-times higher than those reported) (Tondo et al. 2019)

## High dose lithium [> 0.6mmol/L]

Toxicity is usually considered most likely to manifest at levels from 1.5 mmol/L, while concentrations > 2.0mmol/L are associated with potentially life-threatening toxicity. Studies have long reported high serum levels in a sizeable proportion of patients: In one inpatient study, almost 7% of 2210 participants had serum levels of ≥ 1.5mmol/L (1990–1996) but even at this level, less than one third had clinical indications of toxicity (Webb et al. 2001). In a more recent Swedish population-based cohort of patients treated with lithium (1997–2013), the incidence of lithium levels ≥ 1.5mmol was reported to be 0.01 per patient per year (7% over the study period). Of 77 patients with high lithium levels reviewed, over one third required intensive care treatment and 13% received haemodialysis, although all recovered with limited apparent chronic impact on renal function (Ott et al. 2016). Management strategies for toxicity are available (Murphy et al. 2023).

Early studies of acutely manic patients reported antimanic efficacy at 1.3 or 1.4mEq/L (Prien et al. 1972; Spring et al. 1970), with higher doses conferring more toxicity without efficacy, though one further study reported a dose-response effect up to 1.2mmol/L (Stokes et al. 1976). Updated management guidelines for bipolar disorder still report that there may be increased efficacy at levels 0.8–1.0mmol/L (with claims of this effect to 1.0 to 1.5 mmol/L), however extreme caution is recommended, with advice to permit these doses in specific unusual circumstances (e.g., no alternative treatment) and for a short period of time (Goodwin et al. 2016). Support for high-dose lithium is also pertinent for suicidality (Cipriani et al. 2013) and aggressive behaviour in other populations (Malone et al. 2000).

In contrast to acute mania is maintenance prophylactic treatment with lithium for people with bipolar disorders; for this, the landmark analysis by Nolen & Weisler from “Trial 144” identified higher effectiveness with lithium levels ≥ 0.6mmol/L (Nolen and Weisler 2013). A recent meta-analysis supports this although suggests that higher levels up to 1.2mmol/L are primarily effective against depression as opposed to mania relapse risk (Hsu et al. 2022). Notwithstanding these findings, 0.6mmol/L is most commonly considered the optimal trade-off between effectiveness and safety for this indication (Nolen et al. 2019).

### ‘Therapeutic’ dose lithium [0.6-0.8mmol/L]

It is noted that toxicity can occur even at therapeutic doses (as per treatment guidelines for maintenance in bipolar disorder, as its primary indication), although clearly less likely than with higher doses (Goodwin et al. 2016). To firstly discuss the safety considerations with ‘therapeutic’ dose lithium, the greatest concern is typically related to renal function in the long term. While lithium does in some cases adversely affect renal function (particularly older people on long-term therapy (Tondo et al. 2017), the risk of chronic renal illness may not be significantly increased in an overall lithium-treated group (Bosi et al. 2023). An in-depth recent study reported high interindividual variation but steeper declines in estimated glomerular filtration rate (eGFR) explained by lithium use but also found wrongful clinical attribution of some chronic kidney disease (CKD) cases to lithium (Fransson et al. 2022) which may have increased other records-based studies’ estimates (Strawbridge and Young 2022). Relatedly, long-term lithium use can also induce nephrogenic (but not central) diabetes insipidus (Grünfeld and Rossier 2009) and more innocuously, polyuria (Gitlin 2016). The other main concern is with thyroid disorders, which are common, particularly in long-term treated patients (Joseph et al. 2023). While renal risks appear related to long-term (> 10 years) lithium use, thyroid concerns may occur during the first years of treatment (Joseph et al. 2023) and not show a marked increase over subsequent years (Kraszewska et al. 2015). There is also evidence to suggest that, although lithium appears to affect thyroid stimulating hormones, substantive hypothyroidism risk may not be specific to lithium but rather may be a common risk in people with bipolar disorders (Kraszewska et al. 2019). Other concerns at this dose level can include tremor, nausea, fatigue, hyperphagia, increased white blood cell count and hypercalcemia, although many of these are transient and/or can be managed using other strategies (Kovacs et al. 2022; Tondo et al. 2019).

Efficacy for prophylaxis in BD has already been discussed, but the largest meta-analysis to date also demonstrates efficacy in the shorter term for both depression and mania (Ulrichsen et al. 2023). Despite a small evidence base, a recent meta-analysis examining rapid cycling bipolar disorder patients illustrate efficacy relative to other mood stabilisers for depression and global outcomes (Strawbridge et al. 2022). Lithium at this dose is also recommended as an augmentation strategy for non-responsive (Scott et al. 2023) or treatment-resistant (Strawbridge et al. 2019) unipolar depression. The literature on suicidality suggests that lithium can have anti-suicide effects at this dose and thus may be clinically indicated even in mood disorder patients experiencing a limited clinical effect on depression/mania (Tondo et al. 2019). From promising early evidence in cognition studies (Matsunaga et al. 2015), an ongoing trial is examining ‘therapeutic dose’ lithium for the prevention of dementia onset (“LATTICE”).

### ‘Low dose’ [0.2-0.6mmol/L]

Evidence is accumulating to support the utility of ‘sub-therapeutic’ lithium doses. Indeed, many of the trials intending to achieve a ‘therapeutic’ dose of lithium report high proportions of patients with lower serum levels, which has been reported to reduce effect sizes (Nierenberg et al. 2013) but can also highlight areas of benefit despite low doses.

The support for lithium in MCI/dementia suggests that there may not be a significant dose effect: a 2015 meta-analysis, containing trials of doses ranging from 300ug (micrograms) to 168 mg, has reported similar efficacy in high versus low dose studies (Matsunaga et al. 2015). These doses are converted to estimated quantities of *ionic* lithium, with a starting dose of 600 mg lithium carbonate containing 112.5 mg ionic lithium. There is some consensus that low dose lithium could be used to prevent as well as treat cognitive deterioration (Masson et al. 2014). Our systematic review of ‘sub-therapeutic’ lithium effects on various neuropsychiatric outcomes not only identified a consistent effect in benefitting cognitive health in older adult populations, but also found putative (although inconsistent) benefits for depressive and manic symptoms: 2/5 studies of (bipolar and unipolar) depressed participants reported improvements after lithium augmentation; a further study reported benefits to depression (but not multiple sclerosis [MS] symptoms) in participants with MS. For mania, studies generally reported reduced efficacy compared to standard-dose lithium, but single studies reported no difference in remission rates between standard- and low-dose lithium, or compared to carbamazepine while two further studies reported no benefit over placebo or usual care. The following effects were all additionally identified in single studies: improvements to (pseudo)manic symptoms were reported in alcohol use disorder, global impression in dementia (particularly apparent in delusion, irritability and mood lability), and positive and negative symptoms in people at risk for psychosis. In terms of suicidality, one study reported no effect and another identified some benefits to suicidality (unipolar depression). Of the 16 included studies, reports were unanimous safety of low-dose lithium across all studied populations (Strawbridge et al. 2023b). Other studies have reported fewer side effects with doses in this range compared to higher doses, and although it has been suggested that this may not lead to fewer treatment discontinuations (Nolen et al. 2019), it may be that adherence is improved in the absence or reduction of side effects (Barroilhet and Ghaemi 2020). There is a paucity of research into adverse effects of low-dose lithium, although we also cite an as-yet unpublished study finding increased (although clearly non-significant) CKD rates in people with serum lithium levels of 0.3–0.59 (Gíslason et al. 2023).

### ‘Micro’ dose [5-20 mg ionic lithium]

5-20 mg lithium (equivalent to 27-107 mg lithium carbonate) can be purchased over the counter as a nutritional supplement, most commonly in the form of lithium orotate (Strawbridge and Young 2022). No studies of these lithium supplements have formally assessed neuropsychiatric outcomes (Strawbridge et al. 2023b) although two small human studies, conducted decades ago, reported extremely positive findings of lithium orotate (for various diagnoses) notwithstanding notable methodological limitations (Nieper 1973; Sartori 1986). The putative utility, however, of microdose lithium include: (1) safety, given its use in the community for decades without safety concerns (Strawbridge and Young 2022), (2) bioavailability, with some animal studies even suggesting an orotate anion may be able to facilitate relatively higher serum levels than other formulations (Pacholko and Bekar 2023) and preliminary evidence of clinical effects of lithium at similar doses: two of the sixteen studies included in our low-dose lithium systematic review used doses of 300ug (dementia) and 400ug (mood), both reporting effects similar to other studies of somewhat higher doses (Strawbridge et al. 2023b). The evidence is, of course, scant. Given the above, we would posit that microdose lithium may have potential in three particular areas: firstly, in people with age-related cognitive decline (see above), secondly alongside other mood stabilisers in those who have benefitted clinically from high-dose lithium but suffered severe adverse physical effects (Strawbridge and Young 2022), and/or thirdly, for other outcomes if consumed on an ongoing period for an extended period of time (see below). Survey data from 211 people taking microdose lithium purchased commercially suggest that people commonly find benefits to mood, anxiety and cognition (each rated by > 20% of people) with mood commonly reported as the greatest benefit, most frequently ‘moderate’ in magnitude (Strawbridge 2023). While microdose lithium may have potential, we argue that initial feasibility and subsequent substantive examination in clinical trials are warranted.

### ‘Trace’ dose [< 5 mg ionic lithium]

Elemental lithium is naturally present in most rocks, meaning that trace levels are found in mineral water and food grown in soil. This varies substantially across region, with some mineral waters containing up to 9 mg/L (Schrauzer 2002). As a result, average dietary lithium intake has been estimated to increase to as much as 80 µg Li/kg-day (Moore 1995). Furthermore, lithium levels in the general population are measurable with high sensitivity assays e.g., lower limit of detection 0.002ug/L; average level 0.96 µg/L in one study (Enderle et al. 2020). Although heralded as a ‘micronutrient’ for its health benefits, these are yet to be fully established (Szklarska and Rzymski 2019), however, one study found over 8% of the variance in lithium levels could be attributed to diet, with higher lithium levels in people consuming more fruit, vegetables, tea, beer and wine; higher lithium levels were also found in those consuming more alcohol overall, older participants and those with lower eGFR. The authors, however, considered that higher lithium in older participants with low eGFR may indicate reverse causality (Enderle et al. 2020). Indeed, dietary lithium has been associated with improved renal outcomes after transplant (Post et al. 2023)(48). Better known in our field is the increasingly-documented association between higher trace elemental lithium intake from drinking water supplies and reduced suicide rates from ecological studies; this was meta-analysed from areas in Japan, Greece, USA, Austria, UK, Italy and Lithuania (Memon et al. 2020), with similar subsequent studies in Argentina (López Steinmetz et al. 2021), Hungary (Izsak et al. 2022) and Lithuania (Liaugaudaite et al. 2021). Similar, albeit less frequent and/or consistent, reports have been published related to reduced hospital admissions, dementia rates, depressive and anxiety symptoms and violent behaviour (Eyre-Watt et al. 2021) as well as all-cause mortality and premature death (Fajardo et al. 2018). While these findings have not yet been established as associated with confounding factors, most examinations are at a population-level and findings remain uncertain as to individual benefits. There is slightly more support for extrapolation to individuals for dementia rates (Kessing et al. 2017). It is worth noting that a prolonged period of exposure is considered a contributing factor to trace lithium benefits, although the dose/duration required has not been established. We emphasise that as the doses lower in this article, the strength of evidence and consequent certainty of effects diminishes, as do dose windows (particularly between ‘micro’ and ‘trace’ quantities.

## Going down, going forward

In the clinical use of lithium, primarily for affective disorders but also potentially for dementia and transdiagnostically for suicidality or aggression, one message is clear: *“go as low as you can”* without clinical ill effects and make use of the several guidelines to manage adverse effects. A few facts here need emphasising: firstly, the substantial individual differences in absorption of lithium which are known to be affected by several factors such as age (Tondo et al. 2019), caffeine and nicotine (Frigerio et al. 2021). Of course, clinical severity and complexity alongside concomitant interventions are also critical. The focus of intervention in treatment versus prevention is also important, regardless of indication. For some indications, particularly cognitive illness, suicidality and aggression (but also apparent in mood), there is some evidence of effects even with miniscule quantities of lithium but the evidence of micro- and trace-dose lithium is lacking in quantity and rigor. We would urge large-scale, long-term clinical trials of low-dose lithium in the areas where there is most potential, which may be in prevention of significant cognitive decline, suicidality, and/or mood disorders in prodromal or high-risk populations. Comparisons between different formulations (e.g., lithium carbonate versus supplement forms such as lithium orotate) in terms of serum level and dose will also be important. The authors consider that the availability of lithium in such an extensive range of quantities, in combination with its wide-ranging effects, suggest that there is an under-utilised opportunity to fully understand it’s benefits at doses where there are not safety concerns.

## Data Availability

Not applicable.

## References

[CR1] Barroilhet SA, Ghaemi SN (2020). When and how to use lithium. Acta Psychiatr Scand.

[CR2] Bauer M, Gitlin M, Bauer M, Gitlin M (2016). Neuroprotection and other clinical indications of Lithium. Essential guide to Lithium Treatment.

[CR3] Bosi A, Clase CM, Ceriani L, Sjölander A, Fu EL, Runesson B (2023). Absolute and relative risks of kidney outcomes Associated with Lithium vs Valproate Use in Sweden. JAMA Netw Open.

[CR4] Cipriani A, Hawton K, Stockton S, Geddes JR (2013). Lithium in the prevention of suicide in mood disorders: updated systematic review and meta-analysis. BMJ Br Med J Publishing Group.

[CR5] Enderle J, Klink U, di Giuseppe R, Koch M, Seidel U, Weber K (2020). Plasma Lithium levels in the General Population: a cross-sectional analysis of metabolic and dietary correlates. Nutrients.

[CR6] Eyre-Watt B, Mahendran E, Suetani S, Firth J, Kisely S, Siskind D (2021). The association between lithium in drinking water and neuropsychiatric outcomes: a systematic review and meta-analysis from across 2678 regions containing 113 million people. Aust N Z J Psychiatry.

[CR7] Fajardo VA, Leblanc PJ, Macpherson REK. Examining the relationship between Trace Lithium in drinking Water and the Rising Rates of Age-Adjusted Alzheimer’s Disease Mortality in Texas. J Alzheimers Dis 61(1):425–34. 10.3233/JAD-170744 PMC7592673 29103043

[CR8] Fransson F, Werneke U, Harju V, Öhlund L, Lapidoth J, de Jonsson M (2022). Kidney function in patients with bipolar disorder with and without lithium treatment compared with the general population in northern Sweden: results from the LiSIE and MONICA cohorts. Lancet Psychiatry Elsevier.

[CR9] Frigerio S, Strawbridge R, Young AH (2021). The impact of caffeine consumption on clinical symptoms in patients with bipolar disorder: a systematic review. Bipolar Disord.

[CR10] Gíslason G, Indriðason OS, Sigurðsson E, Pálsson R. Risk of development of chronic kidney disease in individuals on lithium therapy, a survival analysis. Personal communication; unpublished findings; 2023.

[CR11] Gitlin M (2016). Lithium side effects and toxicity: prevalence and management strategies. Int J Bipolar Disord.

[CR12] Gomes FA, Brietzke E, Bauer M, Post RM (2022). A call for improving lithium literacy among clinicians and patients. Int J Bipolar Disord.

[CR13] Goodwin G, Haddad P, Ferrier I, Aronson J, Barnes T, Cipriani A (2016). Evidence-based guidelines for treating bipolar disorder: revised third edition recommendations from the British Association for Psychopharmacology. J Psychopharmacol.

[CR14] Grünfeld J-P, Rossier BC (2009). Lithium nephrotoxicity revisited. Nat Rev Nephrol.

[CR15] Hidalgo-Mazzei D, Mantingh T, Pérez de Mendiola X, Samalin L, Undurraga J, Strejilevich S (2023). Clinicians’ preferences and attitudes towards the use of lithium in the maintenance treatment of bipolar disorders around the world: a survey from the ISBD Lithium task force. Int J Bipolar Disord.

[CR16] Hsu C-W, Tsai S-Y, Tseng P-T, Liang C-S, Vieta E, Carvalho AF (2022). Differences in the prophylactic effect of serum lithium levels on depression and mania in bipolar disorder: a dose-response meta-analysis. Eur Neuropsychopharmacol.

[CR17] Izsak B, Hidvegi A, Balint L, Malnasi T, Vargha M, Pandics T (2022). Investigation of the association between lithium levels in drinking water and suicide mortality in Hungary. J Affect Disord.

[CR18] Joseph B, Nunez NA, Pazdernik V, Kumar R, Pahwa M, Ercis M (2023). Long-term Lithium therapy and thyroid disorders in bipolar disorder: a historical cohort study. Brain Sci.

[CR19] Kessing LV, Gerds TA, Knudsen NN, Jorgensen LF, Kristiansen SM, Voutchkova D et al. Association of lithium in drinking water with the incidence of dementia. JAMA Psychiatry Oct;74(10):1005–10. 10.1001/jamapsychiatry.2017.2362 PMC5710473 28832877

[CR20] Kovacs Z, Vestergaard P, Licht W, Straszek RPV, Hansen S, Young ASH (2022). Lithium induced hypercalcemia: an expert opinion and management algorithm. Int J Bipolar Disord.

[CR21] Kraszewska A, Chlopocka-Wozniak M, Abramowicz M, Sowinski J, Rybakowski JK (2015). A cross-sectional study of thyroid function in 66 patients with bipolar disorder receiving lithium for 10–44 years. Bipolar Disord.

[CR22] Kraszewska A, Ziemnicka K, Jończyk-Potoczna K, Sowiński J, Rybakowski JK (2019). Thyroid structure and function in long-term lithium-treated and lithium-naïve bipolar patients. Hum Psychopharmacol Clin Exp.

[CR23] Liaugaudaite V, Naginiene R, Raskauskiene N, Mickuviene N, Bunevicius A, Sher L (2021). Relationship between Lithium Levels in drinking water and suicide rates: a nationwide study in Lithuania. Arch Suicide Res off J Int Acad Suicide Res.

[CR24] López Steinmetz LC, López Steinmetz RL, Diaz SL, Godoy JC (2021). Lithium in drinking water, altitude, and suicide rates in rural areas of Argentinean Andes. Spat Spatio-Temporal Epidemiol.

[CR25] Malone RP, Delaney MA, Luebbert JF, Cater J, Campbell M (2000). A double-blind placebo-controlled study of Lithium in Hospitalized Aggressive children and adolescents with Conduct Disorder. Arch Gen Psychiatry.

[CR26] Masson M, Mauras T, Malhi GS. Lithium the magic ion: restoring and preventing? Aust. N. Z. J Psychiatry. 2014;48(9):874–6. 10.1177/0004867414547780 25147294

[CR27] Matsunaga S, Kishi T, Annas P, Basun H, Hampel H, Iwata N (2015). Lithium as a treatment for Alzheimer’s Disease: a systematic review and Meta-analysis. J Alzheimers Dis JAD.

[CR28] McKeown L, Taylor RW, Day E, Shah R, Marwood L, Tee H (2022). Patient perspectives of lithium and quetiapine augmentation treatment in treatment-resistant depression: a qualitative assessment. J Psychopharmacol Oxf Engl.

[CR29] Memon A, Rogers I, Fitzsimmons SMDD, Carter B, Strawbridge R, Hidalgo-Mazzei D (2020). Association between naturally occurring lithium in drinking water and suicide rates: systematic review and meta-analysis of ecological studies. Br J Psychiatry.

[CR30] Moore JA (1995). An assessment of lithium using the IEHR evaluative process for assessing Human Developmental and Reproductive toxicity of agents. IEHR Expert Scientific Committee. Reprod Toxicol Elmsford N.

[CR31] Murphy N, Redahan L, Lally J. Management of lithium intoxication. BJPsych Adv. Volume 29. Cambridge University Press; 2023. pp. 82–91. 2.

[CR32] Nieper HA (1973). The clinical applications of lithium orotate. A two years study. Agressol Rev Int Physio-Biol Pharmacol Appl Aux Eff Agression.

[CR33] Nierenberg AA, Friedman ES, Bowden CL, Sylvia LG, Thase ME, Ketter T (2013). Lithium treatment moderate-dose use study (LiTMUS) for bipolar disorder: a randomized comparative effectiveness trial of optimized personalized treatment with and without lithium. Am J Psychiatry.

[CR34] Nikolova VL, Pattanaseri K, Hidalgo-Mazzei D, Taylor D, Young AH (2018). Is lithium monitoring NICE? Lithium monitoring in a UK secondary care setting. J Psychopharmacol Oxf Engl.

[CR36] Nolen WA, Weisler RH (2013). The association of the effect of lithium in the maintenance treatment of bipolar disorder with lithium plasma levels: a post hoc analysis of a double-blind study comparing switching to lithium or placebo in patients who responded to quetiapine (trial 144). Bipolar Disord.

[CR35] Nolen WA, Licht RW, Young AH, Malhi GS, Tohen M, Vieta E (2019). What is the optimal serum level for lithium in the maintenance treatment of bipolar disorder? A systematic review and recommendations from the ISBD/IGSLI Task Force on treatment with lithium. Bipolar Disord.

[CR37] Ott M, Stegmayr B, Salander Renberg E, Werneke U (2016). Lithium intoxication: incidence, clinical course and renal function – a population-based retrospective cohort study. J Psychopharmacol.

[CR38] Pacholko AG, Bekar LK (2023). Different pharmacokinetics of lithium orotate inform why it is more potent, effective, and less toxic than lithium carbonate in a mouse model of mania. J Psychiatr Res.

[CR40] Post RM (2018). The New News about Lithium: an underutilized treatment in the United States. Neuropsychopharmacology.

[CR39] Post A, Kremer D, Groothof D, Seidel U, Huebbe P, Franssen CFM (2023). Dietary lithium intake, graft failure and mortality in kidney transplant recipients. Nephrol Dial Transplant.

[CR41] Prien RF, Caffey EM, Klett CJ (1972). A comparison of lithium carbonate and chlorpromazine in the treatment of excited schizo-affectives. Report of the Veterans Administration and National Institute of Mental Health collaborative study group. Arch Gen Psychiatry.

[CR42] Puglisi-Allegra S, Ruggieri S, Fornai F (2021). Translational evidence for lithium-induced brain plasticity and neuroprotection in the treatment of neuropsychiatric disorders. Transl Psychiatry.

[CR43] >Sartori HE. Lithium orotate in the treatment of alcoholism and related conditions. Alcohol 1986;3(2):97–100. 10.1016/0741-8329(86)90018-2 3718672

[CR44] Schrauzer GN (2002). Lithium: occurrence, dietary intakes, nutritional essentiality. J Am Coll Nutr.

[CR45] Scott F, Hampsey E, Gnanapragasam S, Carter B, Marwood L, Taylor RW (2023). Systematic review and meta-analysis of augmentation and combination treatments for early-stage treatment-resistant depression. J Psychopharmacol.

[CR46] Severus E, Nolen WA, Bauer M, Lithium (2021). The best current treatment for the well-informed bipolar patient. Bipolar Disord.

[CR47] Spring G, Schweid D, Gray C, Steinberg J, Horwitz M (1970). A double-blind comparison of lithium and chlorpromazine in the treatment of manic states. Am J Psychiatry.

[CR48] Stokes PE, Kocsis JH, Arcuni OJ (1976). Relationship of lithium chloride dose to treatment response in acute mania. Arch Gen Psychiatry.

[CR49] Strawbridge R. Could Lithium Be A Viable Neuroprotective Supplement? 34th WORLD Congr. Montreal, Canada; 2023.

[CR54] Strawbridge R, Young AH (2022). Lithium: balancing mental and renal health. Lancet Psychiatry Elsevier.

[CR50] Strawbridge R, Carter B, Marwood L, Bandelow B, Tsapekos D, Nikolova VL (2019). Augmentation therapies for treatment-resistant depression: systematic review and meta-analysis. Br J Psychiatry.

[CR53] Strawbridge R, Kurana S, Kerr-Gaffney J, Jauhar S, Kaufman KR, Yalin N (2022). A systematic review and meta-analysis of treatments for rapid cycling bipolar disorder. Acta Psychiatr Scand.

[CR51] Strawbridge R, Izurieta E, Day E, Tee H, Young K, Tong CC et al. Peripheral inflammatory effects of different interventions for treatment-resistant depression: a systematic review. Neurosci Appl 2023a Jan 1;2:101014. 10.1016/j.nsa.2022.101014 PMC12244173 40655965

[CR52] Strawbridge R, Kerr-Gaffney J, Bessa G, Loschi G, Freitas HLO, Pires H et al. Identifying the neuropsychiatric health effects of low-dose lithium interventions: a systematic review. Neurosci Biobehav Rev 2023b Jan 1;144:104975. 10.1016/j.neubiorev.2022.104975 36436738

[CR55] Szklarska D, Rzymski P (2019). Is Lithium a Micronutrient? From Biological Activity and Epidemiological Observation to Food Fortification. Biol Trace Elem Res.

[CR56] Tondo L, Abramowicz M, Alda M, Bauer M, Bocchetta A, Bolzani L (2017). Long-term lithium treatment in bipolar disorder: effects on glomerular filtration rate and other metabolic parameters. Int J Bipolar Disord.

[CR57] Tondo L, Alda M, Bauer M, Bergink V, Grof P, Hajek T (2019). Clinical use of lithium salts: guide for users and prescribers. Int J Bipolar Disord.

[CR58] Ulrichsen A, Hampsey E, Taylor RH, Gadelrab R, Strawbridge R, Young AH (2023). Comparing measurements of lithium treatment efficacy in people with bipolar disorder: systematic review and meta-analysis. BJPsych Open.

[CR59] Webb AL, Solomon DA, Ryan CE. Lithium levels and toxicity among hospitalized patients. Psychiatr. Serv. Volume 52. American Psychiatric Publishing; 2001. pp. 229–31. 2. 10.1176/appi.ps.52.2.229 11157124

[CR60] Yatham LN, Kennedy SH, Parikh SV, Schaffer A, Bond DJ, Frey BN (2018). Canadian Network for Mood and anxiety treatments (CANMAT) and International Society for Bipolar Disorders (ISBD) 2018 guidelines for the management of patients with bipolar disorder. Bipolar Disord.

